# Genetic Identification of Brazilian Mammalian Hosts of *Trypanosoma cruzi*: Improving Blood Meal Source Discrimination in Vector-Borne Transmission

**DOI:** 10.3390/pathogens14060579

**Published:** 2025-06-10

**Authors:** Quezia Moura Oliveira, Thaíla Santos Pessanha, Alena Mayo Iñiguez

**Affiliations:** 1 Laboratório de Parasitologia Integrativa e Paleoparasitologia—LPIP, Instituto Oswaldo Cruz, Fiocruz, Avenida Brasil 4365, Rio de Janeiro 21040-360, RJ, Brazil; queziamouraeea@gmail.com (Q.M.O.); thailasantospessanha@gmail.com (T.S.P.); 2 Programa de Pós-Graduação em Biologia Parasitária, Instituto Oswaldo Cruz, Fiocruz, Avenida Brasil 4365, Rio de Janeiro 21040-360, RJ, Brazil

**Keywords:** blood clot, Brazilian fauna, molecular cloning

## Abstract

The detection of food sources of blood-sucking vectors is essential for a better understanding of the hosts, reservoirs, and other fauna that participate in the transmission web of hemoparasites. The molecular identification of triatomine blood meal sources (BMSs) has been shown to be highly sensitive and taxonomically specific when compared to the immunological method. The application of molecular cloning makes it possible to identify multiple BMS species and/or different individuals/haplotypes of the same vertebrate species in a single triatomine specimen. In Brazil, the molecular detection of BMSs is incipient, with insufficient genetic information on the species of animals involved in the transmission of *Trypanosoma cruzi*. In this work, we evaluated the sensitivity and specificity of a molecular approach using molecular cloning for the detection of multiple Brazilian mammalian species. The DNA was extracted from blood clots of 13 species of canids, bats, xenarthral, marsupials, and rodents. Serial proportions were used to formulate mixtures combining taxonomically close (belonging to the same family or order) and taxonomically distant (different families) species. The results showed that GenBank lacks reference sequences for some native species tested, such as the sylvatic rodent, *Necromys lasiurus*, and the wild canid, *Lycalopex gymnocercus,* for *cyt*b and 12S rDNA, and the rodent *Oecomys cleberi* for 12S rDNA. The study also demonstrated that it is possible to detect multiple different species, even for those that are taxonomically close. This approach was proven to be efficient for the detection of species in equal and even in disparate unequal proportions, which could represent complementary information about the diversity of potential hosts of *T. cruzi.* The detection of multiple BMS species in mixed samples provides a more comprehensive and accurate landscape of *T. cruzi* transmission in nature.

## 1. Introduction

American trypanosomiasis (AT), an infection caused by the *protozoan Trypanosoma cruzi* Chagas, 1909 (Kinetoplastida, Trypanosomatidae), is primarily a wild enzootic that is widely distributed in the mammalian fauna of Brazilian biomes [1]. In humans, this infection can cause Chagas disease (CD), which was discovered in 1909 by the Brazilian physician Carlos Chagas and is still considered today a neglected disease that can result in a debilitating and often fatal chronic condition [2]. It is estimated that about eight million people are infected with CD in Latin America, and more than seventy million are at risk of contracting the infection [3]. In Brazil, the prevalence of CD varies from 1.0 to 2.4%, equivalent to 1.9 to 4.6 million people infected with *T. cruzi* [4].

*Trypanosoma cruzi* can potentially infect all mammalian species, which play significant roles in the transmission cycle [5]. In Brazil, although more than 700 mammalian species have been described based on morphological data, genetic information is only available for a limited number [6]. This limitation hinders genetic studies for species identification, as many of them lack curated reference data. Certain mammals are frequently infected by *T. cruzi*, have been recognized as reservoirs of the parasite [5], and serve as food sources for the triatomine vector.

Brazilian marsupial opossums (Didelphimorphia) are classic reservoirs of *T. cruzi* and other trypanosomatids in the wild [7]. *Trypanosoma cruzi* was isolated and detected by PCR in the species *Didelphis albiventris* (Lund, 1840) and *Monodelphis domestica* (Wagner, 1842) found in the Caatinga region [8,9]. Infections in opossums are well-documented, and their parasite–host interactions have been extensively studied [7,8,10]. Bats (Chiroptera) have a long evolutionary history with trypanosomatids, and it has been postulated that they are the ancestral hosts responsible for introducing trypanosomatid species into the New World [11]. *Trypanosoma cruzi* infection has been reported in more than 20 species across all Brazilian biomes [8,12,13,14,15]. Rodents (Rodentia) are important hosts of *T. cruzi,* and some species represent the main protagonists in the transmission cycle in Northeast Brazil [16]. Synanthropic and wild rodents have been found to be infected in all Brazilian biomes of the country [8,9,17]. Wild and domestic canids (Carnivora) are commonly found infected, despite domestic dogs maintaining low parasitemia in Brazil [18]. Armadillos (Cingulata) are classic and ancient hosts, and their role as a wild reservoir was first defined in the discovery of CD by Dr. Carlos Chagas [2]. All these orders of mammals have been indicated as blood meal sources (BMSs) for *T. cruzi* vectors [16,19,20,21,22,23].

Siqueira (1960), using the immunological technique called precipitin, was the first researcher to demonstrate that it is possible to determine the BMSs of triatomines [24]. The precipitin test and Enzyme-Linked Immunosorbent Assay (ELISA) are based on an antigen–antibody reaction using sera from known mammals, birds, and reptiles, resulting in the formation of a precipitate when combined [24]. However, it is important to note that both tests have taxonomic limitations, providing results only up to the order or family level [25,26]. This leads to low sensitivity and specificity, failing to reflect the actual diversity of BMSs in hematophagous vectors. On the other hand, the molecular identification of BMSs proved to be highly sensitive and taxonomically specific compared to immunological methods. In addition, molecular cloning allows the identification of multiple BMS species and/or different individuals/haplotypes of the same species in a single triatomine specimen [6,7,8].

Studies of the BMSs of hematophagous insects have been applied in epidemiological surveys of infectious diseases in order to better understand the ecology of vectors and hosts, reservoirs, and other non-host environmental fauna that participate in the transmission of hemoparasites.

Recent molecular studies have improved our understanding of host diversity in the *T. cruzi* transmission network [16,22,27,28,29]. Using this approach, species of mammals, birds, and reptiles have been suggested to play different roles in the transmission networks of *T. cruzi* in populations of the *Triatoma brasiliensis* complex from the Brazilian semiarid region, State of Rio Grande do Norte [16,30]. Almeida and collaborators (2016) [28] suggest that rodents are the main BMSs, with distinct roles, such as *Kerodon rupestris* (Wied-Neuwied, 1820) in nature and *Galea spixii* (Wagler, 1831), potentially connecting *T. cruzi* networks in the wild and peridomestic ecotopes. It is known that different profiles of BMSs are consistent with local wild and domestic fauna. Furthermore, variations in blood meal preferences in some vector species, even in sympatry, were suggested [28].

Despite recent studies on the BMSs of triatomines, the deficiency of genetic information available in GenBank for wild mammals involved in *T. cruzi* transmission has been reported [28,31]. This absence of genetic data was observed in the study by Almeida and collaborators (2016) [28], in which the identification of some BMSs was taxonomically limited to the genera *Proceratophrys* and *Mabuya*. The difficulty in the taxonomic identification of species was also observed by Valença-Barbosa and collaborators (2022), showing the family Mustelidae as BMSs [31]. The cut-off for detecting BMSs at the species level was only considered when sequences presented a coverage higher than 97% and genetic identity above 97% and in the 75–97% range for genus/family identification [28].

Considering that BMS studies provide information on a wide diversity of species based on PCR and Sanger sequencing methodologies, the discrimination of multiple BMSs is exclusively obtained by methods such as molecular cloning or next-generation sequencing. From this, the following questions arise: (1) Is there bias in identifying the BMSs with higher proportions? (2) Are the techniques based on clone sequencing efficient at revealing secondary sources with equal or minor proportions? (3) Is the fauna of endemic mammals in Brazil recognized as hosts of *T. cruzi* represented in GenBank? The current work seeks to answer basic methodological questions to enrich the genetic identification of BMSs in the country.

In the current study, we evaluated the sensitivity and specificity of DNA barcoding and molecular cloning for detecting multiple Brazilian mammalian species that are close and distant taxonomically in individual samples. We hypothesized that molecular cloning is effective at discriminating mammal species, even at equal proportions or with a very low secondary representation in the sample.

## 2. Materials and Methods

### 2.1. Blood Clot Samples to Evaluate the Specificity and Sensitivity of the BMS Molecular Approach

To evaluate the specificity and sensitivity of the BMS molecular targets, blood clots from 14 species of mammals involved in the transmission of *T. cruzi* DNA were selected (Table 1). The dataset includes species from five orders—*Didelphis albiventris, Didelphis aurita* (Wied-Neuwied, 1826)*, Monodelphis domestica*, *Marmosa demerarae* (O. Thomas, 1905) (Didelphimorphia), *Canis lupus familiaris* (Linnaeus, 1758), *Cerdocyon thous* (Linnaeus, 1766)*, Lycalopex gymnocercus* (G. Fischer, 1814) (Carnivora), *Oecomys cleberi* (Locks, 1981), *Necromys lasiurus* (Lund, 1841), *Rattus rattus* (Linnaeus, 1758 (Rodentia), *Desmondus rotundus* (E. Geoffroy, 1810), *Sturnira lilium* (É. Geoffroy, 1810), *Carollia perspicillata* (Linnaeus, 1758) (Chiroptera), and *Dasypus novemcinctus* (Linnaeus, 1758) (Cingulata)—from 3 Brazilian biomes, including the Atlantic Forest, Pampa, and Cerrado. Mammal identification and blood clot collection procedures were conducted by veterinary professionals as part of previously performed fieldwork [32]. Blood clot collection was authorized by the Brazilian Institute of the Environment and Renewable Natural Resources (IBAMA) license no. 19037–1 and no. 10070–2.

### 2.2. DNA Extraction from Blood Clots

**Figure 1:**
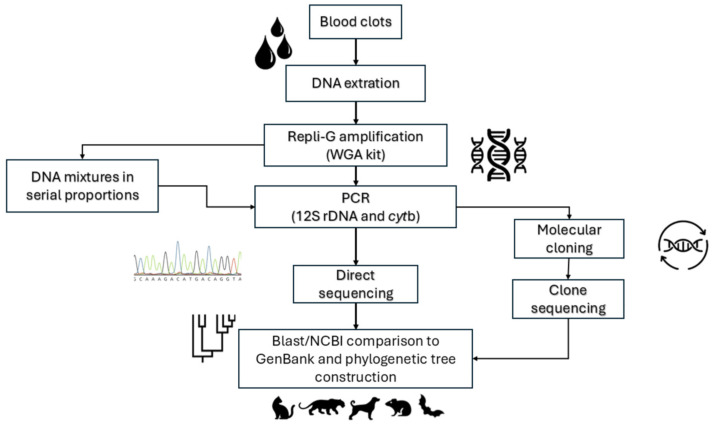
Diagram illustrating the flow of the methodological procedures applied in this study for the use of blood clots from different mammals through vertebrate BMS barcoding using both direct sequencing and clone sequencing approaches.

The flow of the methodological procedures is described in Figure 1. Blood clots were collected and stored in ethanol. DNA was extracted based on the ammonium acetate precipitation protocol [14]. The absolute ethanol was removed, and then the sample was centrifuged at 17,900× *g* for 10 min in buffer (38 mM NaCl, 10 mM EDTA, 5 mM Tris-Cl) to discard any residual ethanol. The supernatant was removed, and the pellet was resuspended in 200 μL of Digsol buffer (120 mM NaCl, 20 mM EDTA, 50 mM Tris-Cl, 1% SDS) and 20 μL of proteinase K at 20 mg/mL. The reaction was incubated in a thermo-shaker at 55 °C for 3 h. After the incubation, 400 μL of 4 M ammonium acetate was added. The DNA was resuspended in 25 μL of buffer (10 mM Tris-HCl pH 7.4; 1 mM EDTA, pH 8.0) and stored at −20 °C until molecular characterization.

### 2.3. Molecular Identification of Mammalian Species and T. cruzi Infection

The detection of mammalian species was based on the DNA barcoding approach with the application of the mitochondrial DNA targets *cyt*b (358 bp) and 12S rDNA (215 bp) [33,34]. The PCR method was applied, with a final volume of 25 µL, including High Fidelity Buffer [600 mM Tris-SO_4_ (pH 8.9), 180 mM (NH_4_) 2SO_4_], 2.5 mM MgSO_4_, 2 mM each dNTP, 2.0 U of Platinum^®^ *Taq* DNA Polymerase High Fidelity (Invitrogen, Paisley, Scotland), and 40 ng of DNA. To check if these blood clots were molecular positive for *T. cruzi* infection and their DTUs, PCR assays were performed based on the 18S rDNA and *cyt*b molecular targets (200 pb) [35,36]. Positive (PCRs using *Mus musculus* DNA) [37] and negative (PCRs without DNA) PCR controls were always included.

PCR products were subjected to agarose (2%) gel electrophoresis, stained with GelRed (Biotium, Inc., Fremont, CA, USA), and visualized under UV light. Amplicons were purified using the Gel Band Purification Kit (GE Healthcare, Chicago, IL, USA), according to the manufacturer’s instructions. DNA direct sequencing was conducted using the Big Dye Terminator v 3.1 Cycle Sequencing Kit (Applied Biosystems, Foster City, CA, USA) and the ABI 3730 sequencer (Applied Biosystems, Waltham, MA, USA) at the RPT01A/FIOCRUZ sequencing facility. Sequences were deposited in GenBank (accession numbers: PV023314-PV023326).

Molecular cloning was performed using the pGEM^®^-T Easy Vector Systems protocol (Promega, Madison, WI, USA). Cloning products were sequenced as described above (Figure 1). All sequences obtained were submitted for editing, alignment, and visualization using Lasergene SeqMan^TM^ v.7.0 (DNASTAR, Madison, WI, USA) and Bioedit v.7.0.5 (Department of Microbiology, North Carolina State University, Raleigh, NC, USA) [38,39]. BLAS TNsearches were performed at NCBI (http://blast.ncbi.nlm.nih.gov/Blast.cgi (accessed on 31 May 2025)) to identify mammalian species using the cut-off values of 97% query coverage and 98% genetic identity. Phylogenetic trees were constructed using the MEGA v.11 software package, utilizing the Neighbor-Joining (NJ) and Maximum Likelihood (ML) methods. Statistical support for the branches was generated by 500 bootstrap replicates.

## 3. Results

### 3.1. Evaluation of the Specificity of Mammalian Species Identification Using BMS Molecular Targets and T. cruzi Infection

All blood clots were negative for *T. cruzi* infection and positive for the two BMS molecular targets *cyt*b and 12S rDNA (Figure S1). Regarding the molecular identification of the mammalian species, it was observed that there are no sequences available in GenBank for the wild canid field fox *Lycalopex gymnocercus* or the wild mouse *Oecomys cleberi* for the two molecular targets *cyt*b and 12S rDNA (Table 2). In addition, *Necromys lasiurus* (Pixuna) did not have 12S rDNA sequences available in GenBank (Table 2). The 12S rDNA and *cyt*b markers demonstrated specificity in species discrimination in all the orders except for didelphids (Table 2). Marsupials of the genus *Didelphis* showed high intra-genus genetic proximity, showed no 12S rDNA genetic distance between *D. aurita* and *D. marsupialis*, and 1.84% genetic distance between *D. aurita* and *D. albiventris,* the species selected for this study (see below). Therefore, we decided not to perform *Didelphis* intra-genus mixing.

The 12S rDNA *D. aurita* sequence revealed a maximum genetic identity of 99.08% with the only two *D. aurita* sequences available in the database and, importantly, with the *D. marsupialis* sequence (NC057518). In addition, the *D. aurita* sequence obtained showed high genetic identity with *D. albiventris* at 98.16%. For the *cyt*b marker, the *D. aurita* sequence obtained displayed a maximum genetic identity of 99.40% for the available *D. aurita* sequences. There are fourteen *cyt*b sequences in the database for this species. *Didelphis aurita* showed 98.50% genetic identity with *D. marsupialis* and 96.40% with *D. albiventris.* Species that are not distributed in Brazil but showed a high genetic similarity with *D. aurita* in this study were *D. pernigra* (NC057519) at 97% and *D. virginiana* (NC001610) at 95.09%.

The 12S rDNA sequences generated from *D. albiventris* in this study revealed a maximum genetic identity of 99.36% with *D. albiventris* from GenBank (Table 2). There are only two sequences deposited in the database for this marker. *Didelphis albiventris* also showed high genetic identity with three other species of the same genus: *D. imperfecta* (NC057517) at 98.73% and both *D. marsupialis* and *D. aurita* at 98.09%. For the *cyt*b target, the *D. albiventris* sequence had 99.85% maximum identity with the available *D. albiventris* data. *Didelphis albiventris* also exhibited a high genetic identity with three other species of the same genus: *D. perniga* with 96.26% genetic identity and *D. marsupialis* and *D. aurita* both with 95% genetic identity.

The *Marmosa demerarae* 12S rDNA sequence obtained presented a genetic identity of 99.05% with *M. demerarae*. It also showed 97.14% identity with *M. murina* (KX381772). For the *cyt*b target, the *M. demerarae* sequence had a higher genetic identity of 98.80% with *M. demerarae*. Other species of the same genus were also identified: *M. murina* with 97.90%, *M. limae* (MN978639) with 97.31%, and *M. constantiae* (MK496216) with 96.47%. The 12S rDNA sequences generated by the *Monodelphis domestica* pointed to a maximum genetic identity of 97.36% with the available *M. domestica* sequences. For the *cyt*b target, the *M. domestica* sequence had a higher genetic identity of 100% with *M. domestica* (Table 2).

The 12S rDNA sequence of the *Lycalopex gymnocercus* canid species showed a maximum genetic identity of 98.63% with *Lycalopex sechurae* and with other canid genera: 97.26% genetic identity with *Speothos venaticus* (NC053974), 96.80% with *Chrysocyon brachyurus* (KJ508409), and 95.89% with *Canis lupus familiaris*. The *cyt*b sequence of *L. gymnocercus* also displayed a maximum genetic identity of 99.06% with *L. sechurae*, and with canids *Speothos venaticus* at 97.64%, and *C. l. familiaris* at 95.17%. One wild canid sample selected from a cloth bank as *Cerdoncyon thous* was identified as *L. sechura* based on 12S rDNA (Table 2). Therefore, a misidentification of the blood clot, probably with *L. gymnocercus*, was presumed. The sample was omitted from the *cyt*b analysis and molecular cloning procedure. The *C. l. familiaris* sequence of the 12S rDNA target demonstrated the highest genetic identity of 100% with at least one thousand sequences of the species.

The *Necromys lasiurus* rodent sequence of the 12S rDNA marker showed a maximum genetic identity of 97.70% with *Akodon montensis*. There are no sequences available in the database for the *Necromys* genus. *Neotoma floridana* (AF294340) species, from a different genus, was also identified with 95.79% genetic identity. For the *N. lasiurus cyt*b target, the maximum genetic identity was 98.57% with the species *Bolomys lasiurus* and 95.14% with *B. temchuki* (AY273913) and *Oligoryzomys fornesi* (EU192158). The 12S rDNA *Rattus rattus* sequence presented 100% genetic identity with *R. rattus* sequences. The species *Sundamys infraluteus* (NC036728) was also identified with 98.17% genetic identity. The *cyt*b sequence had the highest genetic identity of 99% with the first one hundred *R. rattus* sequences deposited in the database. The *Oecomys cleberi* rodent 12S rDNA sequence obtained a maximum genetic identity of 98.92% with *O. bicolor*. There are no sequences deposited for this marker in the database for *O. cleberi*. Other species were also identified: *Oecomys auyantepui* (KX381574) with 97.85% genetic identity and *Hapalomys delacouri* (MZ159976), *Euryoryzomys macconnelli* (KX381426), and *Acomys cahirinus* (JN571145) all with 96.76% genetic identity. For the *cyt*b marker, *O. cebleri* had the highest genetic identity of 99.16% with *O. cleberi* and 98.88% with *O. bicolor*.

The 12S rDNA sequence of the chiropteran *D. rotundus* pointed to a maximum genetic identity of 97% with *D. rotundus* and 90.60% with another bat species, *Choeronycteris mexicana*. For the *cyt*b target, a sequence obtained from *D. rotundus* revealed the highest genetic identity of 99% with the available *D. rotundus* sequences, and the second highest identification with *Diaemus youngii* (FJ155475), with 89.05% genetic identity. The 12S rDNA sequence of the chiropteran *Sturnira lilium* showed the maximum identity of 100% with *S. lilium*, 99.17% with *S. magna* (AY395845), and 98.60% with *S. tildae* (NC022427). For *cyt*b, the *S. lilium* sequence presented 100% genetic identity with *S. lilium* and 99.3% with *S. tildae* (NC022427). The 12S rDNA sequence of *Carollia perspicillata* showed maximum identity with *C. perspicillata*, 99.07% with *C. bravicauda* (NC066073), 98.14% with *C. costanea* (NC065677), and 96.74% with *Micronycteris nicefori* (AY395830). The *C. perspicillata cyt*b sequence had 100% genetic identity with *C. perspicillata* for the first one hundred sequences available for this genetic marker.

The 12S rDNA sequence of the xenarthral *Dasypus novemcinctus* had the highest genetic identity of 100% with *D. yepesi*; 99.53% with the three species *D. novemcinctus, D. sabanicola* (NC028568), and *D. bellus* (MW963147); and 99.07% with the three species *D. septemcinctus* (KT818546), *D. hybridus* (NC028565), and *D. guianensis* (PP916004). There are only nine sequences available in GenBank for this marker. For the *cyt*b target, the sequence of *D. novemcinctus* had a maximum identity of 99% with *D. novemcinctus*, 98.73% with *D. yesepi* (NC028570), and 92.72% with *D. pilosus* (NC028567).

**Figure 2:**
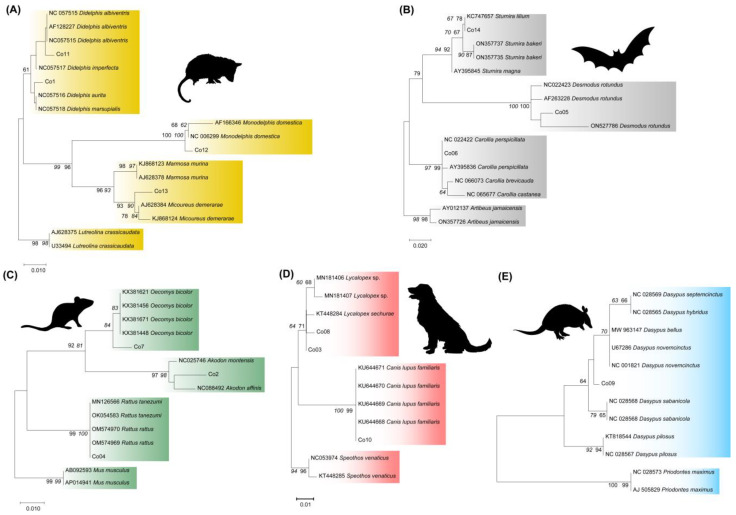
ML phylogenetic tree for the 12S rDNA target from the blood clots recovered from Brazilian mammals. (**A**) Opossums: CO01 = *Didelphis aurita*; CO11 = *Didelphis albiventris*, CO12 = *Monodelphis domestica*, CO13 = *Micoureus demerarea*. (**B**) Chiropterans: CO05 = *Desmodus rotundus*, CO06 = *Carollia perspicillata*, CO14 = *Sturnira lilium* (**C**) Canids: CO 03 and 08 = *Lycalopex schurae,* CO10 = *Canis lupus familiaris.* (**D**) Armadillo: CO09 = *Dasypus novemcinctus*. (**E**) Rodents: CO02 = *Akodon montensis*, CO04 = *Rattus rattus*, CO07 = *Oecomys bicolor.* The tree was constructed in MEGA v.11 software using ML and NJ methods and the Kimura 2-parameter model plus the gamma distribution with 500 bootstrap replicates. Reference sequences are indicated by species name and the GenBank numbers. The species used as outgroups were (**A**) the opossum *Lutreolina crassicaudata* (Desmarest, 1804); (**B**) chiropteran *Artibeus jamaicensis* Leach, 1821; (**C**) rodent *Mus musculus* (Linnaeus, 1758); (**D**) canids *Speothos venaticus* (Lund, 1842); and (**E**) armadillo *Priodontes maximus* (Kerr, 1792). Bootstrap values are shown on the internal nodes as ML = regular font and NJ = italic font.

NJ and ML phylogenetic trees for each order were constructed using the sequences obtained from the 14 species and their best-scoring sequences in GenBank. Both reconstructions showed the same topology. The ML phylogeny that includes the Didelphimorphia species from the present study showed three genus-specific clusters: *Didelphis* spp., *Monodelphis* spp., and *Marmosa* spp. (Figure 2A). The sequences obtained from *Monodelphis domestica* (CO12) and *Marmosa demerarea* (CO13) were grouped into each genus cluster and were closely related to their corresponding GenBank species reference with high bootstrap values (Figure 2A). However, the *Didelphis* spp. cluster was moderately supported, and the sequences from *D. aurita* (CO01) and *Didelphis albiventris* (CO11) were poorly defined (Figure 2A), confirming our option of not conducting serial mixtures between these species.

The Chiropteran phylogenetic tree demonstrated that the *D. rotundus* (CO05), *C. perspicillata* (CO06), and *S. lilium* (CO14) sequences were located in each corresponding genus clade and were closely related to the GenBank species reference with high bootstrap values (Figure 2B).

The Rodentia phylogenetic reconstructions showed *R. rattus* (CO04) grouping in a species cluster (Figure 2C). However, *Oecomys cleberi* (CO07) was located in an *O. bicolor* species cluster, and *Necromys lasiurus* (CO02) grouped into an *Akodon* spp. clade (Figure 2C), reflecting the absence of these native species in the GenBank dataset, which limits accurate species-level mammalian identification and could consequently produce an incomplete and mistaken interpretation of the scenario of host–parasite transmission.

The ML 12S rDNA tree from canids (Figure 2D) showed two main well-defined clusters, one from *Canis lupus familiaris*, which includes the CO10 sequence, and a second one from the *Lycalopex* genus (Figure 2C). With the absence of a GenBank deposit of *L. gymnocercus* (CO08), the species sample (CO8) grouped into the *Lycalopex* genus clustered with the *L. sechura* sequence. The canid cloth sample named *Cerdoncyon thous* (CO3) clustered with *L. sechura*. Therefore, the misidentification of the blood clot sample with *L. gymnocercus* was confirmed. Finally, the Armadillo 12S rDNA phylogenetic analysis revealed the *Dasypus novemcinctus* (CO09) sequence near a cluster that included *D novemcinctus* reference sequences (Figure 2E).

### 3.2. Sensitivity Assessment of Detecting Multiple Blood Clot DNAs

**Figure 3:**
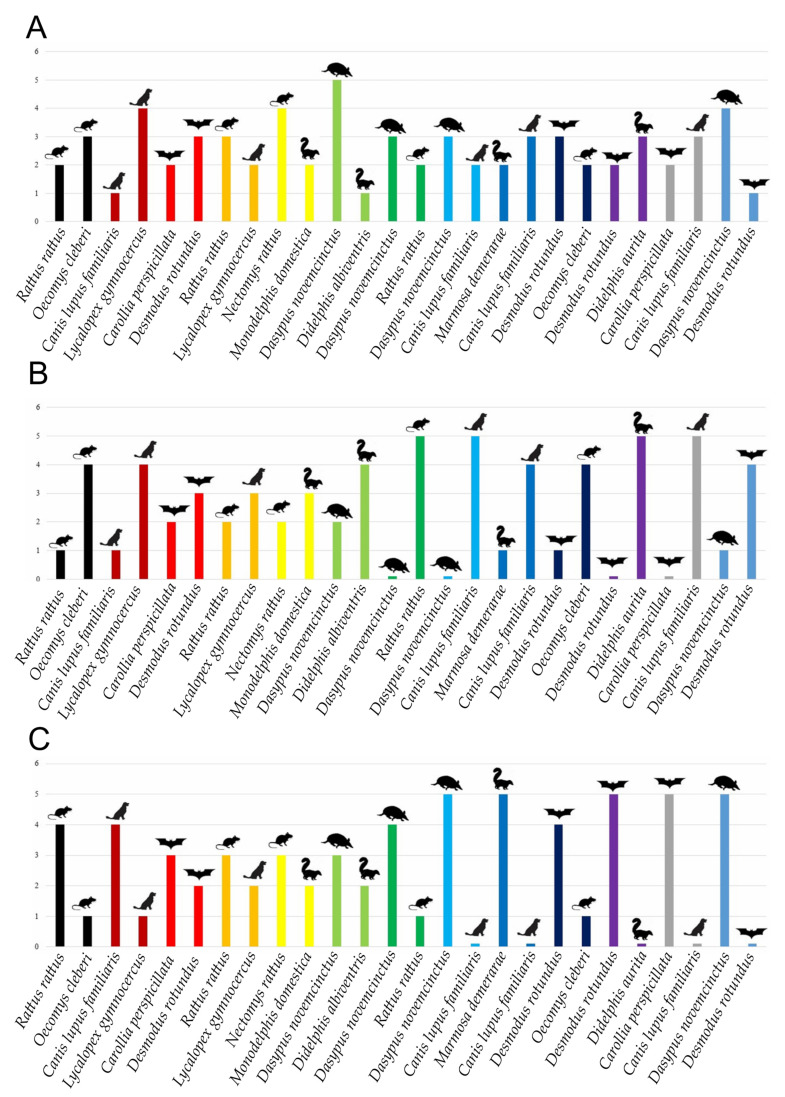
Number of clones detected from 39 multiple mixtures of 13 mammalian species’ DNA. (**A**) Number of clones detected in the proportion of 5/5. (**B**) Number of clones detected in the proportion of 1/9 (**C**). Number of clones detected in the proportion of 9/1. Bars in the same color show the 13 mixtures performed. The animal symbols represent the orders, and the names of species included in each intrafamilial and interfamilial mixture are shown.

Clone sequencing throughout molecular cloning demonstrated the detection of all 13 species used in the 13 multiple mixtures in equal proportions (5/5), including those that are close and those that are unrelated taxonomically (Figure 3). It was possible to detect the species in a lower concentration in 17/26 multiple mixtures of mammalian species in disparate proportions (Figure 3B,C). In the proportions of 1/9 and 9/1, the species in the lower proportion were detected in 9/13 mixtures and 8/13 mixtures, respectively. All 13 species with a higher proportion were detected in the mixed samples, as expected. A total of 9/26 species with lower proportions in multiple mixtures were not detected (Figure 3B,C).

## 4. Discussion

### 4.1. Specificity of Molecular Species Identification

Identifying triatomine BMSs increases our understanding of the hosts involved in the transmission cycle of *T. cruzi* in nature. However, this analysis is usually performed using the precipitin technique, which has low sensitivity and specificity and does not permit an observation of the feeding eclecticism of these insect vectors. Moreover, knowledge of potential BMSs is essential, as this technique detects specific antibodies in the blood that respond to the animal protein origin.

On the other hand, with the application of molecular biology techniques, such as PCR and Sanger sequencing using DNA barcoding, it is possible to identify the BMSs at the species level. However, this method relies on a robust and complete dataset of the fauna from the area of the *T. cruzi* transmission network studied. There is a lack of available sequences for species in GenBank, particularly for mammals native to Brazil [16,22,28].

In the current work, we first evaluated the DNA barcoding approach in discriminating species of mammals involved in the transmission of *T. cruzi* in Brazil. It was observed that the BMS molecular markers 12S rDNA and *cyt*b demonstrated specificity in the discrimination of the species from the orders selected, except for didelphids. Opossum species *Didelphis aurita* and *D. albiventris* showed a close genetic distance (less than 1%). This genetic identification has already been observed previously by Steiner et al. (2015), who identified the phylogenetic relationships of 19 species of *Didelphis* using two nuclear markers, the non-coding transthyretin intron 1 (TTR) and the inter-photoreceptor coding retinoid-binding protein exon 1 (IRBP), and the same two mitochondrial genes that were used in the present study, *cyt*b and 12S rDNA [40].

For the marsupial species *M. demerae,* the subgenus *Micoureus* has already been clarified in the literature by Voss and Jansa (2009), even though the authors mentioned the possibility of keeping *Micoureus* as a genus due to the monophyly of the group [41]. It was possible to observe an inter-genus genetic distance between *M. demerarae* and *M. domestica,* and between the *Monodelphis* and *Didelphis* genera [41]. The high genetic distance among these genera has already been reported by Agrizzi and collaborators (2005), who constructed a phylogenetic tree using *cyt*b gene sequences [42].

Regarding the canid species from the present study, the genetic distance considering the reference sequences of GenBank does not show genetic proximity among them. This was previously shown by Fuentes-González and Muñoz-Durán (2012), who studied the phylogenetic relationships of 35 canid species [43]. *Canis lupus familiaris* and *C. lupus* have a low genetic distance for both molecular markers used in this study. Studies using mitochondrial DNA markers demonstrated genetic proximity between these species, and it was suggested that *C. lupus* is the only ancestor of *C. l. familiaris* [44,45]. Even though *C. lupus* and *C. l. familiaris* are genetically close species, the question of whether they are two distant species has been discussed [45]. The molecular markers showed specificity in discriminating the canid species. The blood clot labeled as *C. thous* was identified as *L. gymnocercus* through the sequence analysis. Thus, this species was excluded from the mixture analysis in the present work due to the incorrect name of the blood clot sample.

It is known that a lack of reference sequences deposited in GenBank can lead to the incorrect identification of species. In BMS studies, this lack can cause the erroneous association of species that contribute to the *T. cruzi* life cycle. In the present work, *L. gymnocercus* GenBank deposits for both markers were absent, generating a maximum identity of the Brazilian field fox with *L. sechurea*, which is a species that does not occur in Brazil. This canid species inhabits the coastal deserts of northern Peru and southern Ecuador [44]. According to the Taxonomic Catalog of the Fauna of Brazil (Catálogo Taxonômico da Fauna do Brasil—http://fauna.jbrj.gov.br/fauna/listaBrasil (accessed on 31 May 2025), two *Lycalopex* species occurred in the country, *L. gymnocercus* and *L. vetulus* (Lund, 1842), both absent from GenBank. Therefore, we strongly recommend targeted sequencing efforts for absent and underrepresented species in the GenBank genetic database, some of which have been repeatedly highlighted in the present study.

The same situation was observed for the lack of an *O. cleberi* 12S rDNA reference sequence in the database, which generated genetic identification with other species of the genus, such as *O. bicolor*, *O. auyantepui*, and other rodent genera like *Hapalomys delacouri*, *E. macconnelli*, and *A. cahirinus*. *Oecomys auyantepui* is only found in the Brazilian Amazon biome [46], *O. catherinae* occurs in the Brazilian Atlantic Forest and Cerrado biomes [47], and *O. bicolor* occurs in the Pantanal, Cerrado, and Amazon biomes [48]. According to Musser and Carleton (2005), the genus *Oecomys* presents an unclear taxonomy [49]. This was confirmed by Suárez-Villota and collaborators (2017), who studied phylogenetic relationships and the delineation of *Oecomys* species using multiple cytogenetic, morphological, and molecular (mtDNA) analyses. The authors concluded that species of the genus *Oecomys* form a species complex and highlighted the importance of combining cytogenetics, morphology, and geographic information with molecular analyses in the delimitation of species [42]. The taxonomic revision of this group was also suggested by Rocha and collaborators (2015) [50]. *Euryoryzomys macconelli* occurs in the Brazilian Amazon biome, and the genetic distance from the *Oecomys* genus has already been demonstrated [51].

The 12S rDNA *Necromys lasiurus* sequence obtained in the current study was identified by BLAST/NCBI as *Akodon montensis*. No sequences of the *Necromys* genus are deposited for this target in GenBank. *Necromys lasiurus* and *A. montensis* belong to the same subfamily Sigmodontinae [52,53], which could be a reason why *A. montensis* appears as a BLAST/NCBI result. Using the *cyt*b target, a sequence of *Bolomys lasiurus* presented 99% genetic identity with the *N. lasiurus* from this study. According to the Integrated Taxonomic Information System, *B. lasiurus* is currently an invalid species, a synonymous species of *N. lasiurus.* In this sense, we highlight the importance of taxonomically updating the GenBank deposits to avoid incorrect nomenclature and missing synonymy information. This failure could raise unrealistic ecological premises about the number and role of species serving as BMS, and whether the blood-feeding behavior of vectors is broad or restricted, with critical implications for understanding vector–host relationships.

It is important to mention that the three rodent species studied in this work do not present genetic proximity, as can be seen in the rodent phylogenetic study carried out by Vilela and collaborators (2014) [54].

The three chiropteran species selected for this work, *C. perspicillata*, *D. rotundus*, and *S. lilium*, showed high genetic distances among them, as can be seen in the work by Baker and Hoofer (2003), who performed a phylogenetic analysis of different genera of bats using mtDNA [55].

The bat species *Sturnira lilium* and *S. tildae* showed genetic distances of 1.4% and 0.7% for 12Sr DNA and *cyt*b, respectively. The genus *Sturnira* is characterized as a group that contains complex phylogenetic relationships [56,57]. Due to their genetic similarity, *S. tildae* and *S. lilum* were suggested to be congeneric species [57] but not sister species [58].

In the present study, *C. perspicillata* and *C. brevicauda* showed high genetic proximity, but this can be explained because *C. perspicillata* and *C. brevicauda* are sister species and closely related to *C. castanea* [59]. The difficulty in identifying these species based only on morphological characters was reported [55].

The Xenarthra species *D. novemcinctus* showed high genetic proximity with *D. yepesi* for the 12S rDNA marker and with *D. novemcinctus* for the *cyt*b marker. The high genetic proximity of these species was shown by a systematic analysis using the *cyt*b marker [60]. The species *D. yepesi*, which showed the highest genetic identity with *D. novemcinctus*, is not considered a valid species by the Integrated Taxonomic Information System (ITIS). We found evidence of a synonymy issue with *D. mazzai* (Yepes, 1933), a valid species that occurs in northern Argentina, but has no genetic sequences deposited in GenBank for any gene [61]. These genes are widely used for the identification of BMSs, and their absence directly affects the accurate identification of the species acting as a BMS, thereby impairing the understanding of transmission dynamics.

It is important to mention that birds and reptiles, considered refractory to *T. cruzi* infection, also served as vector BMSs. However, the current evidence shows that these animals can be infected by *T. cruzi*, and, specifically, reptiles were proposed as silent hosts of the parasite [62,63]. This type of research should be stimulated to provide a larger and more diverse dataset of the Brazilian fauna, not only for BMS studies but also for other enzootic diseases, biodiversity studies, biogeography, taxonomy, and molecular systematics.

### 4.2. Sensitivity in Detecting Multiple DNA Samples from Blood Clots

DNA barcoding studies for the identification of triatomine BMSs are restricted to the detection of a single BMS, which limits the spectrum of potential species that participate in the parasite transmission network or in the maintenance of vector colonies. The application of this approach and subsequent molecular cloning allows the discrimination of multiple BMS species and/or different individuals of the same species by a haplotype analysis [64,65].

In the present study, the molecular cloning analysis demonstrated superior detection of the species with higher proportions, as expected. However, it was possible to detect species with lower concentrations in the different mixtures performed. For mixtures of the same proportion, both species were always detected. Relevant species present in smaller proportions were detected in 54% of the mixtures. From these results, it is possible to state that gene cloning using the 12S rDNA target is effective at detecting multiple BMSs and individuals of the same taxon, even in extremely unequal proportions. The specificity of the 12S rDNA target in the detection of DNA from vertebrate animals has previously been commented [64], and it has even been mentioned that this target is superior for identifying degraded samples compared to the *cyt*b target. The sensitivity of the cloning technique for detecting multiple BMSs has already been mentioned in previous works [64,65]. However, this technique was only applied once previously in triatomine BMS studies in Brazil [36]. The next-generation sequencing method is also efficient at retrieving multiple BMSs in a single vector [66]. However, in comparison, molecular cloning is low-cost, relatively short, and less time-consuming, and does not require very specialized operators to perform the technique and to process the results.

### 4.3. The Importance of the Specificity and Sensitivity in Recovered Mammalian DNA for a Better Understanding of the Life Cycle of T. cruzi

The role of marsupials in maintaining the life cycle of *T. cruzi* is already well supported because these mammals share habitats with the triatomine vectors and feed on these insects and on reservoir species, such as small rodents, which can potentially be infected [10]. Legey and collaborators (1999) highlighted that some didelphids are important reservoirs of *T. cruzi* due to their ability to control the infection and not present clinical changes [67]. Some marsupial species, such as *D. aurita* and *D. marsupialis*, circulate in different ecotopes and can assist in the dispersion of the agent in sylvatic, domestic, and peridomestic environments [10,67]. In addition to moving between different ecotopes, marsupials can use all parts of the forest, being in contact with the different transmission cycles of *T. cruzi* [5]. The omnivorous diet of marsupials favors oral transmission by the predation of infected triatomines or other small mammals. Another marsupial species that was also identified as the main reservoir of *T. cruzi* in an outbreak of Chagas disease in Brazil was *M. domestica* [68]. *Marmosa demerarae* was found to be infected by the parasite [69]. The xenarthral species *D. novemcinctus* was identified as the second most important reservoir of *T. cruzi* after *D. marsupialis* [70].

In the domestic environment, *C. l. familiaris*, the domestic dog, is also reported as a major reservoir host of *T. cruzi* and as a risk factor for human infection [71,72,73]. Dogs may live inside houses and wander through wild ecotopes where they can acquire *T. cruzi* infection from sylvatic triatomines and/or infected prey. These mammals that circulate between domestic and wild ecotopes play very important roles in connecting the transmission network of the parasite [74]. In general, canids can maintain the *T. cruzi* infection throughout their lives [75].

Wild canids are part of a poorly studied taxon in terms of their possible roles in the transmission cycle of *T. cruzi* in nature [76]. These mammals are known to explore large areas and different habitats and can be considered parasite bioaccumulators, especially those that can be transmitted orally, such as *T. cruzi* [76]. This characteristic of wild canids to explore different ecotopes is an extremely critical aspect of the dispersion of the parasite [77].

Rodents are considered the main prey animals that circulate in peridomestic ecotopes [68]. These small mammals connect sylvatic, peridomestic, and domestic ecotopes, transporting the parasite to human dwellings, where they are transmitted to humans and domestic animals. For these reasons, these hosts are important in the study of *T. cruzi* transmission [78].

Almeida and coauthors (2024) [21] proposed that for the transmission cycle of the parasite to occur and lead to CD, five interactions are necessary: parasite, vectors, hosts, environments, and human action. The study highlights the role of humans in producing artificial domestic and peridomestic environments, such as the accumulation of wood piles, which favor the colonization of vectors and small mammals [21].

*Rattus rattus*, a synanthropic animal, has been suggested as a *T. cruzi* reservoir that connects different ecotopes [79]. The authors also mentioned that the proximity of these species to the domestic environment favors the contact of the parasite with the human population. The wild species of rodent, *O. cleberi*, was previously reported to be infected by *T. cruzi* with a high diversity of trypanosomatid species [80]. On the other hand, *N. lasiurus* is one of the most abundant species in the Brazilian Cerrado and is considered a significant host of *T. cruzi* in the biome [8].

For bats, *D. rotundus* and *C. perspicillata* were recently identified as reservoirs of *T. cruzi* in the Atlantic Forest biome [13]. This was not the first report in which bats were considered reservoirs of *T. cruzi*. In Colombia, one of the main food sources detected in triatomines infected by *T. cruzi* was bats [81]; in Ecuador, chiropter was identified as being responsible for the connection between the wild and household ecotopes [82]; in Argentina, *D. rotundus* was found to be infected with *T. cruzi* [83]; and in Venezuela, bats were identified as dispersers of the parasite [84]. The application of the NGS technique reinforced the roles of bats as hosts/reservoirs and as bioaccumulators of *T. cruzi* [13].

## 5. Conclusions

The present study aimed to respond to some basic questions regarding the BMS molecular methodology. The first question concerned the possibility of a bias in identifying BMSs with higher proportions. This was answered since we showed that species with very low proportions of 1/9 and 9/1 were detected in 17/26 of mixed samples, although species with higher proportions were identified in all 26 mixtures.

The second question concerned whether cloning sequencing is efficient at revealing secondary sources or equal proportions of BMSs. Our results demonstrated the detection of two mammalian species in equal proportions in all 26 mixed samples, and, as mentioned above, the secondary or low representative species were also recovered in 17/26 mixed samples. We are aware that the low concentration detection in mixed samples was partial, with a number of false-negative results (9/26). Future efforts to enhance assay sensitivity are necessary to address the false-negative results.

Finally, we questioned if the fauna of endemic mammals in Brazil recognized as hosts of *T. cruzi* are represented in GenBank. Despite the limited number of mammalian species evaluated, 3/13 species did not have GenBank sequences available for the two BMS molecular markers applied.

Thus, the study provides more detailed information on multiple BMS detection protocols that could reflect a succession of vector contact events, the diversity of mammalian hosts, and the complexity of the parasite transmission web.

Therefore, the data confirmed our hypothesis that molecular cloning is effective at distinguishing mammalian species, even with equal proportions or with a very low secondary representation in the sample. The integration of these methodologies in the detection of multiple BMSs could provide more detailed information that could reflect a series of vector contact events, the diversity of mammalian hosts, and the complexity of the parasite transmission network. Future research should further amplify the analysis of more Brazilian mammalian species and explore these dynamics in the wild to obtain effective information about the ecology of *T. cruzi* transmission in nature.

## Figures and Tables

**Table 1:** Data regarding the samples collected from the Brazilian mammalian species for the present study.

Mammalian Species	Common Name in Brazil	Collection Site ^1^	Brazilian Biome
*Canis lupus familiaris*	Domestic dog	Mangaratiba-RJ	Atlantic Forest
*Cerdocyon thous*	Bush dog	Mamamguape-PB	Atlantic Forest
*Lycalopex gymnocercus*	Field fox	Alegrete-RS	Pampa
*Oecomys cleberi*	Tree mouse	Cumari-GO	Cerrado
*Necromys lasiurus*	Pixuna	Mamamguape-PB	Atlantic Forest
*Rattus rattus*	Rat	Paraty-RJ	Atlantic Forest
*Desmodus rotundus*	Vampire bat	Mamamguape-PB	Atlantic Forest
*Sturnira lilium*	Fruit bat	Mamamguape-PB	Atlantic Forest
*Carollia perspicillata*	Short-tail bat	Mamamguape-PB	Atlantic Forest
*Didelphis albiventris*	White-ear possum	Mamamguape-PB	Atlantic Forest
*Monodelphis domestica*	Short-tailed cuica	Mamamguape-PB	Atlantic Forest
*Marmosa* (*Micoureus*) *demerarae*	Cuica	Mamamguape-PB	Atlantic Forest
*Didelphis aurita*	Black-ear possum	Mamamguape-PB	Atlantic Forest
*Dasypus novemcinctus*	Armadillo	Mamamguape-PB	Atlantic Forest

^1^ Brazilian States: RJ—Rio de Janeiro, PB—Paraíba, RS—Rio Grande do Sul, GO—Goiás.

**Table 2:** Results of the genetic analysis of mammalian species detection using the 12S rDNA and *cyt*b rDNA BMS molecular markers.

Mammal Species	Blast/NCBI Genetic Identity Result	Maximum Genetic Identity	GenBank Accession Number	Blast/NCBI Genetic Identity Result	Maximum Genetic Identity	GenBank Accession Number
	Molecular Target 12S rDNA			Molecular Target *cyt*b		
***Canis lupus familiaris***	*Canis lupus familiaris*	100%	KU291093	*Canis lupus familiaris*	99%	DQ309764
***Lycalopex gymnocercus***	*Lycalopex sechurae*	98.63%	KT228284	*Lycalopex sechurae*	99.06%	KT447686
***Cerdocyon thous***	*Lycalopex sechurae*	98.00%	KT448284	ND	ND	ND
***Oecomys cleberi***	*Oecomys bicolor*	98.92%	JF693852.1	*Oecomys cleberi*	98.88%	KR190443
***Necromys lasiurus***	*Akodon montensis*	97.70%	KF769456	*Bolomys lasiurus*	98.57%	KR190443
***Rattus rattus***	*Rattus rattus*	100%	OM574970.1	*Rattus rattus*	99.00%	KT232247
***Desmodus rotundus***	*Desmodus rotundus*	97.00%	HG003310	*Desmodus rotundus*	99.00%	HG003310
***Sturnira lilium***	*Sturnira lilium*	100%	KC747657	*Sturnira lilium*	100%	KC747657
***Carollia perspicillata***	*Carollia perspicillata*	100%	HG003309	*Carollia perspicillata*	100%	FJ589715.1
***Didelphis albiventris***	*Didelphis albiventris*	99.36%	NC057515.1	*Didelphis albiventris*	99.85%	AF089802
***Monodelphis domestica***	*Monodelphis domestica*	99.53%	AJ508398	*Monodelphis domestica*	100%	KM071418
***Marmosa (Micoureus) demerarae***	*Marmosa* (*Micoureus*) *demerarae*	99.05%	AJ628384.1	*Marmosa* (*Micoureus*) *demerarae*	98.80%	AJ487006
***Didelphis aurita***	*Didelphis aurita*	99.08%	NC057518.1	*Didelphis aurita*	99.40%	GU112886
***Dasypus novemcinctus***	*Dasypus yesepi*	100%	NC028570	*Dasypus novemcinctus*	99.00%	KU253494

ND: The analysis was not performed.

## Data Availability

All sequences generated in this study have been submitted to the GenBank database under the accession numbers: PV023314-PV023326.

## Supplementary Materials

The following supporting information can be downloaded at: https://www.mdpi.com/article/10.3390/pathogens14060579/s1, Figure S1. Amplification of mammalian DNA in blood clots and electrophoresis on an agarose gel. (A) Amplification of the molecular target 12S rDNA. Lane 1—Promega DNA Ladder 100 bp, Lane 2—CO01 = *Didelphis aurita*, Lane 3—CO02 = *Akodon montensis,* Lane 4—CO03 = *Lycalopex schurae*, Lane 5—CO04 = *Rattus rattus*, Lane 6—CO05 = *Desmodus rotundus*, Lane 7—CO06 = *Carollia perspicillata*, Lane 8—CO07 = *Oecomys bicolor*, Lane 9—CO08 = *Lycalopex schurae*, Lane 10—CO09 = *Dasypus novemcinctus*, Lane 11—CO10 = *Canis lupus familiaris*, Lane 12—CO11 = *Didelphis albiventris,* Lane 13—CO12 = *Monodelphis domestica*, Lane 14—CO13 = *Micoureus demerarea*, Lane 15—CO14 = *Sturnira lilium*, Lane 16—negative control. (B) Amplification of the molecular target *cyt*b. Lane 1—Promega DNA Ladder 100 bp, Lane 2—CO01 = *Didelphis aurita*, Lane 3—CO02 = *Akodon montensis*, Lane 4—CO03 = *Lycalopex schurae*, Lane 5—CO04 = *Rattus rattus*, Lane 6—CO05 = *Desmodus rotundus*, Lane 7—CO06 = *Carollia perspicillata*, Lane 8—CO07 = *Oecomys bicolor*, Lane 9—CO08 = *Lycalopex schurae*, Lane 10—CO09 = *Dasypus novemcinctus*, Lane 11—CO10 *= Canis lupus familiaris*, Lane 12—CO11 = *Didelphis albiventris*, Lane 13—CO12 = *Monodelphis domestica*, Lane 14—CO13 = *Micoureus demerarea*, Lane 15—CO14 = *Sturnira lilium*, Lane 16—negative control.
