# Supplementary figures and images for: Genetic Identification of Brazilian Mammalian Hosts of Trypanosoma cruzi: Improving Blood Meal Source Discrimination in Vector-Borne Transmission
Source: Pathogens. 2025 Jun 10;14(6):579. doi: 10.3390/pathogens14060579 (PMC12195716; doi:10.3390/pathogens14060579)

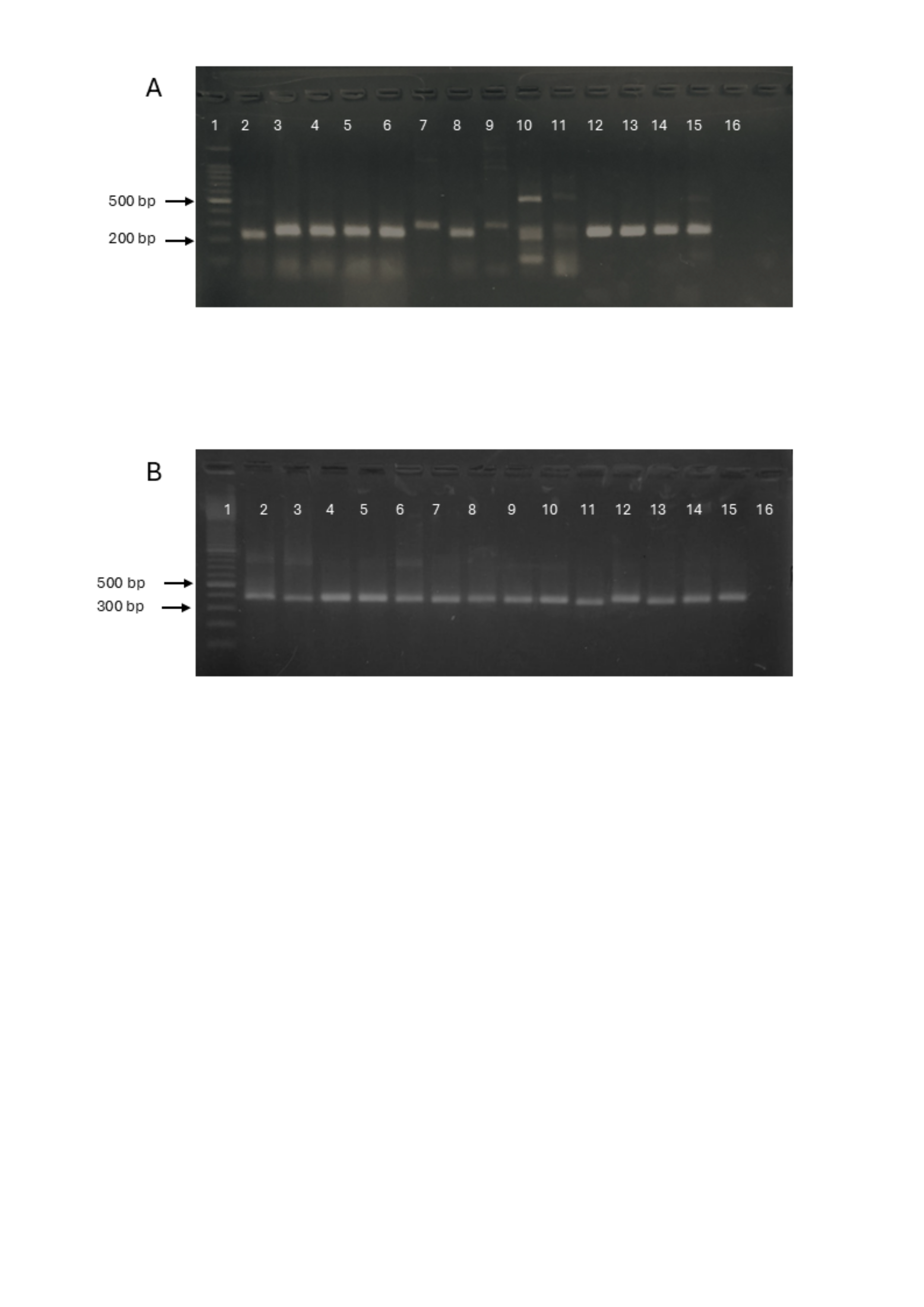

Supplement: Supplementary file 1 [file pathogens-14-00579-s001.zip › Gel Figure S1.png]
